# Health research participants are not receiving research results: a collaborative solution is needed

**DOI:** 10.1186/s13063-017-2200-4

**Published:** 2017-10-02

**Authors:** Christopher R. Long, M. Kathryn Stewart, Pearl A. McElfish

**Affiliations:** 1 College of Medicine, Department of Psychiatry, Division of Health Services Research, University of Arkansas for Medical Sciences Northwest, Fayetteville, AR USA; 2 Fay W. Boozman College of Public Health, University of Arkansas for Medical Sciences, Little Rock, AR USA; 3 College of Medicine, Department of Internal Medicine, University of Arkansas for Medical Sciences Northwest, Fayetteville, AR USA

## Abstract

Health research participants want the results of the studies in which they participate but do not typically receive them. Researchers generally express support for sharing results with participants but, in practice, may be unprepared or unwilling to do so. Many funders call for increased dissemination of research results beyond academic and clinical audiences, but few funders sponsor research to improve result sharing with participants. Although the solution appears straightforward (e.g., funders could incentivize researchers to share results with participants), there are critical gaps in knowledge that suggest the need for a more deliberate approach. For example, what ethical or practical concerns discourage researchers from returning results to participants? What exactly do participants plan to do with the results that they would like to receive? What are the best channels of communication for sharing results with particular participant populations? To address these knowledge gaps, we argue for a collaborative process to develop a research agenda related to result sharing with participants. With support and encouragement by funders, such research should evaluate the effects of different types of results (and results from different types of studies) on participants’ behaviors, attitudes, and emotions; it should also examine the researchers’ ethical, financial, logistical, methodological, and skill-related concerns and constraints related to sharing results with participants. Over time, collaborative research between researchers and participants can yield an evolving set of evidence-based guidelines for ethical, effective result sharing with participants.

## Background

Most health research participants want the results of the studies in which they participate. This has been a consistent finding for more than a decade. A 2008 review of 28 studies found that a median of approximately 90% of health research participants from a wide range of types of studies report interest in receiving results of the studies in which they participate; [1] four new papers report similar findings [2–5].

Despite their clearly expressed preferences, participants do not typically receive the results of the studies in which they participate. In a recent survey of approximately 3400 registrants of ResearchMatch, a large registry of health research participants, only 33.0% of respondents reported receiving results from studies in which they had participated. Instead, 51.8% indicated never having an opportunity to request results, 9.0% elected not to receive results, and 6.2% reported having never receiving results that they had requested [4].

This situation raises ethical concerns, and it may negatively influence participants’ attitudes toward researchers and future research participation. Across many different types of research, participants speak of having a right to the results of the studies in which they have participated [1, 3, 5], and they emphasize the importance of receiving results for themselves, their quality of life, and their families and communities [3, 4].

The problem may not be due to researchers’ reticence to share results. Researchers generally express support for sharing results with participants [1]. In practice, however, many researchers appear to be unprepared or unwilling to do so [1, 4, 6]. Even among community-based participatory researchers, results are not necessarily communicated to participants. A recent review found that only 48% of 101 journal articles describing community-based participatory research studies mentioned dissemination efforts apart from the journal publications themselves [6].

At the same time, many funders—including the United States National Institute on Minority Health and Health Disparities [7] and the Patient Centered Outcomes Research Institute [8]—call for more dissemination of research results beyond academic and clinical audiences; however, despite participants’ clearly stated preferences to receive results, few funders sponsor research to improve sharing results with participants or incentivize researchers to do so.

## Finding a solution: address the knowledge gaps first

The path to a solution appears straightforward: health research participants want to receive the results from the studies in which they participate, and researchers and funders mostly support sharing research results with participants. Moreover, participants say that they want aggregated results that explain the overall findings of the trial more than they want individual results [4]. They are open to receiving results through relatively low-cost channels like email or websites [3, 4]. They are willing to wait until results have been reviewed by other researchers for accuracy and until after the study has been published [4]. They express particular interest in results related to studies’ purposes and any medical advances based on the results [4]. In short, these results are the kinds of aggregate information that researchers compile as they prepare academic publications and presentations. In this way, researchers who would like to share results with participants could capitalize on efforts in which they likely already engage. At this point, one might conclude that the solution is easy and researchers just need to start sharing results, even by sending participants a copy of (or a link to) an academic journal article based on the study. Furthermore, funders could incentivize researchers to share results with participants, and the problem might quickly be solved. However, that solution becomes more complicated when the problem is examined more closely.

Critical gaps in knowledge about result sharing suggest the need for a more deliberate approach. For example, why do researchers express support for returning results to participants but then not return those results to participants? Are researchers choosing not to return results because of ethical concerns (e.g., concerns about how participants will understand or use the results, concerns that the information will induce confusion or negative emotions [1, 9, 10], concerns that results may not generalize [11, 12], etc.)? Or, is result sharing primarily constrained by financial, logistical, methodological, or skill-related barriers?

There is also ambiguity around the best channels of communication through which to share results. Different channels of communication may provide different benefits and drawbacks [2–4]. Participants appear open to modes of communication that may have relatively low cost to researchers (e.g., email or websites) [3, 4], but more interactive approaches (e.g., teleconferences) may assuage researchers’ ethical concerns or improve participants’ willingness to take part in future studies. There is evidence that participants’ communication preferences vary with study topics, the emotional valence of results, and the characteristics of participant populations [3, 5].

Perhaps the greatest unknown with respect to participants’ preferences related to receiving results is what, if anything, participants want to do with those results. Participants often enroll in health research with the expectation of improving their health [13]; it is, therefore, not surprising that they often express a preference to receive results that highlight medical advances or potential clinical implications for themselves or loved ones [1, 4]; however, it is unclear how, or whether, participants intend to act upon results. For those participants who may intend to act, it also unclear how, or whether, to present specific information to contextualize the results, including limitations on the generalizability of results, the need for replication, or comparative effectiveness with other treatments or interventions. If results are to be communicated effectively, participants’ intentions—which likely vary according to participant population, research design, and context—must be better understood.

A final knowledge gap is the extent to which existing knowledge about result sharing generalizes to specific participant populations, research designs, and contexts. What specific harms or benefits are more or less likely to occur when sharing results with participants from different types of research? In what specific ways do barriers and facilitators to sharing results vary as a function of participant populations, research designs, context, etc.? (For example, what are the specific challenges and opportunities associated with sharing results with participants in longitudinal studies?) While the existing academic literature on result sharing spans diverse types of research, conclusions often rely on aggregating findings from very different types of health research or focusing carefully on findings from a single research study in a specific setting with a specific population. Moreover, with some exceptions [14–16], the existing academic literature on result sharing relies heavily upon studies with North American and European participants, and the extent to which conclusions generalize across regions or cultural or ethnic groups is unknown. These uncertainties necessitate caution when applying findings from the existing academic literature on result sharing, and they signal areas that future research in this area must better address.

## Collaboration to improve communication of results

Participants, researchers, and funders appear generally supportive of sharing results with participants. Because these stakeholders have shared interests but unique perspectives on this issue, we advocate for a collaborative process to develop a research agenda to address knowledge gaps related to result sharing.

For participants, research should directly compare the effects of different types of result information on behavior, attitudes, and emotions. Studies using experimental and mixed-methods designs to explore real-time experiences of receiving (or not receiving) results could identify participants’ specific goals with respect to using the information that they receive. Those studies could help researchers and funders gauge the extent to which they should focus on providing recommendations or warnings, contextualizing results, referring participants to specific resources, etc. In other words, this line of investigation could inform researchers and funders with respect to the extent and types of additions or clarifications that should be made to result information that would typically be presented in an academic journal article. Given researchers’ varying resources in terms of personnel time and funding, this line of investigation could conserve resources by focusing efforts to share results on those activities that respond both cost-effectively and efficaciously to participants’ preferences.

To achieve effective result sharing to participants, researchers’ constraints must also be investigated. In addition to understanding and addressing financial, logistical, methodological, and skill-related barriers, it is imperative to understand and address researchers’ ethical concerns related to result sharing [11, 17]. Researchers face the difficult task of minimizing potential negative consequences that may result from knowledge gaps related to (1) which results to share, (2) how best to describe results to participants, and (3) how to reduce participants’ confusion or discomfort related to the results that they receive (or fail to receive) [11]. Institutional Review Board and Ethics Committee policies will heavily influence the development of researchers’ result sharing behavior, and it will be important to study the effects of policies that encourage researchers to formulate plans for result sharing early in the protocol development process, to mention returning results as part of the informed consent process, or to allow every participant to opt in or out of receiving results [18, 19].

Funders can encourage this collaborative effort by funding research on sharing results with participants. By fostering a research agenda that addresses their own preferences alongside those of participants and researchers, funders can help researchers identify best practices for effective result sharing beyond academic and clinical settings. Once these practices have been identified, funders could require—and provide funding for—dissemination efforts that target participants and their communities.

## Conclusions

Only through collaboration between participants (or their representatives) and researchers—ideally with the support of a range of funders—can participants’ preferences, researchers’ constraints, and funders’ priorities be incorporated into the development and implementation of a research agenda to investigate results’ communication, and, eventually, an evolving set of evidence-based guidelines for ethical, effective result sharing with research participants. There are already potential starting points for such a research agenda: The Children’s Oncology Group (COG), a clinical trials group supported by the National Cancer Institute, developed its own recommendations in 2011 for sharing results of COG-funded studies with participants [18]. Based on discussion of empirical and theoretical findings existing in 2011, these recommendations address when, how, and what results should be shared with participants and their parents/guardians. Similarly, the Multi-Regional Center for Clinical Trials assembled researchers, industry representatives, and patient advocates to develop detailed recommendations for returning aggregate results to participants in clinical trials [19]. These recommendations are accompanied by a researcher toolkit with guidance for planning and implementing result sharing [20]. Although focused on COG research and clinical trials, respectively, these two sets of recommendations could serve as a foundation for a research agenda to investigate effective result sharing with a broader group of researchers and participants.

Potential starting points exist for involving participants (or their representatives) in a collaborative research agenda. Patient advocacy groups, public and patient involvement groups, or Community Advisory Boards are accessible for many researchers, and these groups may be able to provide participant-collaborators or community investigators to represent the perspectives of research participants from specific populations.

Sustained collaborative research on best practices for result sharing is necessary to resolve complexities that implicate multiple stakeholders (e.g., to clarify how researchers can best balance participants’ preference to receive results against the understanding that receiving research results can be disturbing or confusing) [1]. Engaging participants, researchers, and funders in a sustained effort to improve results’ communication can increase the likelihood that health research participants (and their families and communities) who would like to receive results will receive them and will receive them in forms that are both useful and respectful of their preferences.

## Abbreviations

COG: Children’s Oncology Group
